# Implementing two national responsibilities of the revised UNICEF/WHO Baby‐Friendly Hospital Initiative: A two‐country case study

**DOI:** 10.1111/mcn.13422

**Published:** 2022-09-29

**Authors:** Altrena Mukuria‐Ashe, Alyssa Klein, Charlotte Block, Kanji Nyambo, Malia Uyehara, George Mtengowadula, Godwin Nyirongo, Adil Mansimov, Samat Okenov, Jeniece Alvey

**Affiliations:** ^1^ USAID Advancing Nutrition Arlington Virginia USA; ^2^ Save the Children USA Washington District of Columbia USA; ^3^ John Snow, Inc. (JSI) Research & Training Institute, Inc. Arlington Virginia USA; ^4^ NCBA CLUSA Washington District of Columbia USA; ^5^ Independent Consultant Lilongwe Malawi; ^6^ SP Trainers & Research Consultants Lilongwe Malawi; ^7^ M‐Vector LLC Bishkek Kyrgyzstan; ^8^ Public Health Institute/USAID Global Health Technical Professionals Washington District of Columbia USA

**Keywords:** Baby‐Friendly Hospital Initiative (BFHI), BFHI national responsibilities, breastfeeding policies, breastfeeding programmes, incentives, Kyrgyz Republic, Malawi, sanctions, technical assistance

## Abstract

The 2018 implementation guidance for the Baby‐Friendly Hospital Initiative (BFHI) recommends institutionalising the ten Steps through nine national responsibilities for universal coverage and sustainability. As countries adapt BFHI programmes to this paradigm shift away from traditional designation programmes, documenting and sharing policy and programme experience are critical and currently sparse. This qualitative case study included desk reviews of published and grey literature on BFHI programming, national plans and policy documents specific to the selected national responsibilities for universal coverage and key informant (KI) interviews across a range of actors. In the Kyrgyz Republic, the case study explored responsibility 5, development and implementation of incentives and/or sanctions, and responsibility 6 in Malawi, providing technical assistance (TA). In both countries, the three sustainability responsibilities (national monitoring [7] communication and advocacy [8] and financing [9]) as they relate to the universal coverage of the targeted responsibilities were also explored. Thirty‐eight respondents in the Kyrgyz Republic described approaches that were used in the health system, including BFHI designation plaques, performance‐based financing and financial sanctions. However, currently, there are no formal incentives and sanctions. In Malawi, TA was utilised for national planning and to introduce quality improvement processes. Forty‐seven respondents mostly described provisions of TA in building and strengthening the capacity of providers. More programmatic evidence to demonstrate which types of incentives or sanctions can be effective and sustained and more documentation of how TA is provided across multiple aspects of implementation are needed as countries institutionalise BFHI.

## Key messages

1. National policies and national and subnational plans need to be effectively communicated throughout the health system and systematic coordination are important to institutionalising Baby‐Friendly Hospital Initiative (BFHI).
2. Technical assistance, commonly provided for professional competency building, could support hospital practices, procedures and management.
3. Understanding the motivations of providers and health systems and monitoring key indicators are important for incentives and sanctions to be effective.
4. Documenting and sharing programmatic experiences are critical as countries adopt the new BFHI guidance.
5. Multisectoral advocacy for the ten Steps is needed to secure national and local commitment and funding across and beyond the health system.

## Graphical abstract

This qualitative case study, conducted during the pandemic in the Kyrgyz Republic and Malawi, reports on country efforts to address two national responsibilities of the 2018 revised UNICEF/WHO Baby‐Friendly Hospital Initiative (BFHI). In the Kyrgyz Republic, lessons learned from previous incentives and sanctions suggest that motivations of the health system and providers need to be considered and the system tailored for BFHI. In Malawi, technical assistance was used to support health provider breastfeeding competency in the absence of in‐service training and could be expanded to support multiple aspects of BFHI implementation.

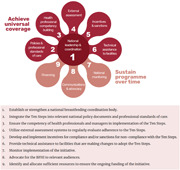

## 1 INTRODUCTION

More than 820,000 deaths among children under age five could be prevented worldwide annually if all children were optimally breastfed. In 1991, the World Health Organisation (WHO) and the United Nations Children's Fund (UNICEF) launched the Baby‐Friendly Hospital Initiative (BFHI) to address the low rates of breastfeeding globally and to motivate health facilities to support optimal breastfeeding through the implementation of Ten Steps to Successful Breastfeeding (Ten Steps) (WHO et al., 1991). Evidence shows that implementation of the Ten Steps improves breastfeeding practices, including improved attitudes and skills of health workers and changes in facility practices to support breastfeeding (Gomez‐Pomar & Blubaugh, 2018; Perez‐Escamilla et al., 2016; WHO, 2017).

**Figure 1:**
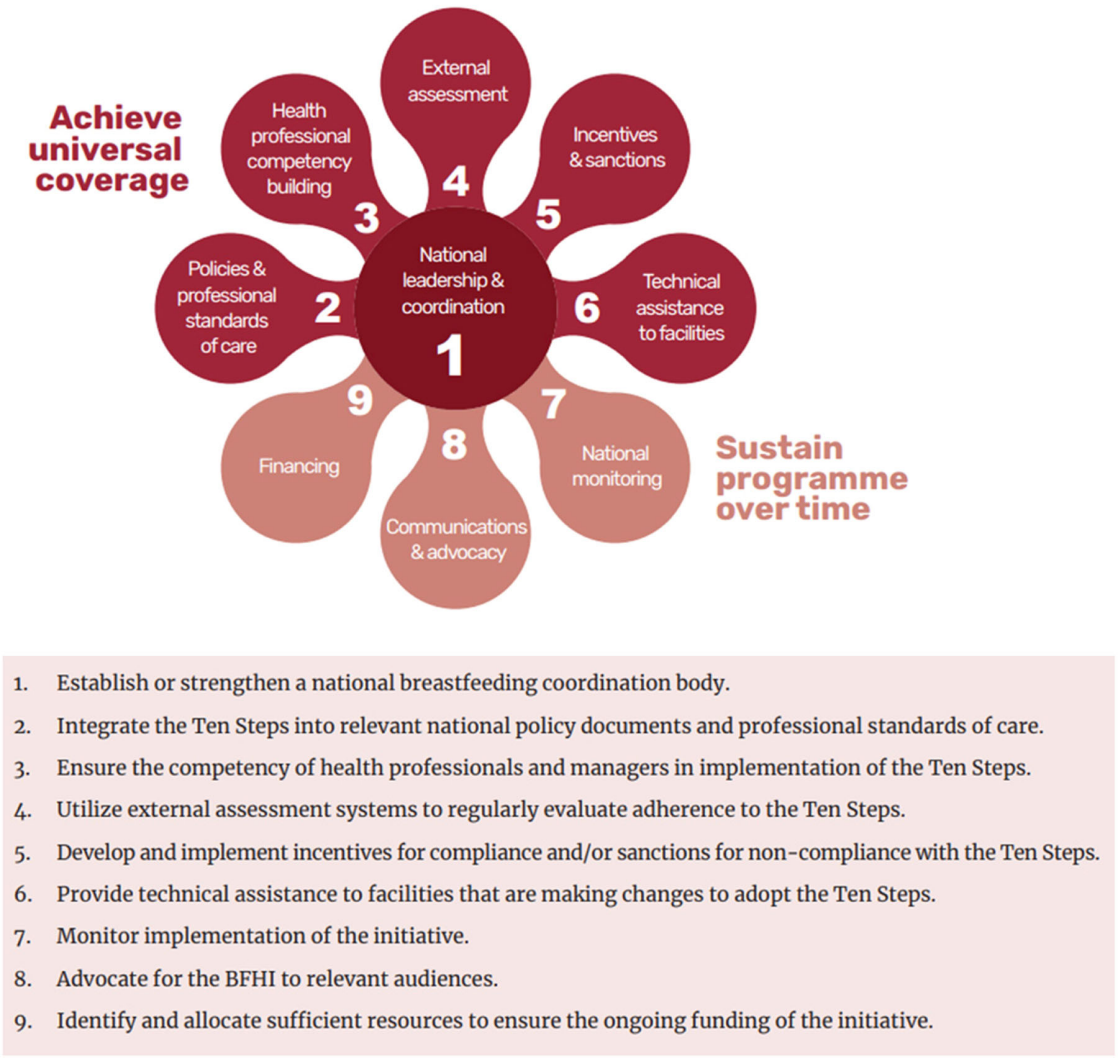
Nine key national responsibilities for BFHI. BFHI, Baby‐Friendly Hospital Initiative. *Source*: WHO (2018, p. 30)

In 2018, WHO and UNICEF updated the Ten Steps to reflect the evidence‐based WHO 2017 guidelines and presented new strategies for national implementation (WHO, 2017; UNICEF &  WHO, 2018). This new implementation guidance recommends a paradigm shift away from traditional Baby‐friendly facility designation to accreditation processes and institutionalisation of the Ten Steps into standards of care and more through nine responsibilities for country implementation of BFHI (Figure 1) (UNICEF & WHO, 2018).

There was little published on how countries are responding and integrating BFHI into standards of care before the COVID‐19 pandemic (UNICEF & WHO, 2017) and hardly any since. USAID Advancing Nutrition, the US Agency for International Development's (USAID) flagship multisectoral nutrition project, led by the John Snow Inc. Research & Training Institute, Inc. (JSI) and a diverse group of partners, conducted this case study in the Kyrgyz Republic and Malawi. Due to limited time and resources, in this paper, we explore how two of the national responsibilities for universal coverage of BFHI are being carried out (Supporting Information: Annex 1). Because of the government‐led results‐based financing scheme for ensuring health care service quality, we chose responsibility #5 for the Kyrgyz Republic. We chose responsibility #6 for Malawi because the government has received and provided technical assistance (TA) for a number of years with partner support. We also explore efforts relevant to three sustainability responsibilities (national monitoring [7], communication and advocacy [8] and financing [9]) as they relate to responsibilities #5 and #6.

### 1.1 Research questions

The case study sought to answer the following guiding questions:

1. What kind of TA are facilities receiving to comply with the Ten Steps? What TA strategies help to build competency and institutionalise the provision of TA to facilities? (Malawi)
2. What incentives or sanctions support compliance with the Ten Steps? What opportunities exist for innovation to motivate facilities to comply with the Ten Steps? (Kyrgyz Republic)
3. What efforts are underway at the national level to achieve universal coverage and sustainability of these two national responsibilities? What challenges were encountered?
4. What lessons learned could strengthen universal coverage and sustainability of BFHI in other countries?

Like many countries, Malawi and the Kyrgyz Republic were reinvigorating their BFHI efforts before the release of the 2018 guidance. These case studies serve as a qualitative snapshot of how the two countries are working towards the selected national responsibilities and may contribute to the necessary paradigm shift for universal coverage and sustainability of implementing the Ten Steps.

### 1.2 Country background

#### 1.2.1 The Kyrgyz republic

The Kyrgyz Republic, a land‐locked, mountainous country located in Central Asia, borders China, Kazakhstan, Uzbekistan and Tajikistan, covering 199,000 sq. km (76,834 sq. mi). With a multiethnic population of almost six million people, the majority of whom live in rural areas, there is a considerable gap in the standard of living between these families and those in the major urban centres (National Statistical Committee of the Kyrgyz Republic NSC Ministry of Health Kyrgyz Republic and ICF International, 2013).

Almost 100% of births occur in a health facility, and 81% of infants are breastfed within 1 hour of birth. In contrast, 46% of children under 6 months are exclusively breastfed. Nearly all antenatal care takes place in health facilities and is provided by a doctor in 84% of cases (National Statistical Committee NSC of the Kyrgyz Republic and UNICEF, 2019).

The Kyrgyz Republic has a strong national policy environment, with more than 20 years of laws, policies and regulations related to breastfeeding best practices (Table 1). In 2000, the Kyrgyz Republic adopted BFHI and certified its first hospital as baby‐friendly. The proportion of medical facilities declared baby‐friendly peaked in 2010 at 76%, but declined to 42% by 2015, mostly due to lack of funding for training and reassessments (UNICEF & WHO, 2017).

**Table 1:** Selection of breastfeeding‐related policies

Year	Title	Description
1996	Order N19	The order supports exclusive breastfeeding in health facilities, including rooming‐in, initiation of breastfeeding within 1 h after birth, breastfeeding exclusively and on demand until 4–6 months of age, and bans feeding infants before lactation.
2000	Adoption of BFHI	The Kyrgyz Republic began working towards BFHI certification in health and medical facilities.
2008	Breastmilk Substitute (BMS) Policy	The policy prohibits the unnecessary use or promotion of BMS in medical facilities.
2009	Order N8	The government adopted an eleventh step to strengthen prohibition of the use of BMS in facilities and added indicators around the Eleven Steps for routine evaluation of standard practices. (The 2019 policy reverts back to Ten Steps and this manuscript references only these ten).
2010	Order N68	The order protects, supports and promotes breastfeeding among infants in maternity hospitals and children's hospitals.
2015	Order N585	The policy on nutrition and food security. The Mother‐Friendly Criteria Initiative was renamed to the Baby and Mother‐Friendly Hospital Initiative.
2019	Order N1078	The order updated and expanded the Ten Steps for comprehensive care for successful breastfeeding in maternity hospitals.

Abbreviation: BFHI, Baby‐Friendly Hospital Initiative.

#### 1.2.2 Malawi

Malawi, a landlocked country in southeastern Africa, borders Tanzania, Zambia and Mozambique, covering 118,760 sq. km (45,853 sq. mi), and is defined by its topography of highlands split by the Great Rift Valley and Lake Malawi. It has a population of 19 million, the majority of whom are engaged in cash‐crop and subsistence agriculture.

In 2017, 61% of children under 6 months were exclusively breastfed in Malawi, a decline from 72% in 2010 (National Statistical Office NSO Malawi and ICF, 2017). Early initiation of breastfeeding stands at 77% (Nkoka et al., 2019). The overall prevalence of facility births is 91%, 51% of women had at least four antenatal visits and 94% of women received antenatal care from a skilled provider (National Statistical Office NSO Malawi and ICF, 2017).

Malawi has a strong policy environment for BFHI. The MOH adopted BFHI into legislation in 1993 in response to an exclusive breastfeeding rate of 3% (National Statistical Office NSO Malawi and ICF, 2017; National Statistical Office NSO Malawi and Macro International, 1994). Table 2 lays out the key BFHI‐related legislation and policies in Malawi. The current National Multi‐Sector Nutrition Strategic Plan (2018–2022) presents BFHI as a key strategy for promoting, protecting and supporting breastfeeding. Investments in vehicles for incentivizing BFHI adherence continue; a new Food and Nutrition Bill before Parliament supports nutrition programming, including BFHI and stronger protection for the International Code of Marketing of Breastmilk Substitutes (the Code) (World Health Organisation WHO, 1981).

**Table 2:** Selection of breastfeeding‐related policies, strategies and actions, Malawi

Year	Title	Description
1993	Malawi launched the WHO/UNICEF BFHI	The Government of Malawi committed to protect, promote and support breastfeeding.
2008	Marketing of Breastmilk Substitutes Policy	The policy was first gazetted in 2004 and updated in 2008. It is part of the documents included in BFHI training for health professionals.
2009	Adopted BFHI	Malawi started to use BFHI as a strategy to promote, protect and support breastfeeding.
2017	Adapted/revised BFHI 20‐hour course manual	Malawi revitalized BFHI to train more people and revised the training manual.
2018	National Multi‐Sector Nutrition Policy 2018–2022	The policy guides the implementation of nutrition interventions, including BFHI.
2019	Multi‐Sector Maternal Infant and Young Child Nutrition Strategy 2019–2023	The strategy provides guidance on infant feeding, including breastfeeding.
2021	Food and Nutrition Bill	Currently before Parliament, this bill would establish a National Nutrition Council with the budget and power to oversee food safety and to set and enforce rules for the promotion and marketing of foods for infants and young children.

Abbreviations: BFHI, Baby‐Friendly Hospital Initiative; WHO, World Health Organisation.

Since adoption of BFHI in 1993 until 2007, 48 of the 644 facilities providing maternity services received training, but the MOH only certified 26 of these hospitals with external funding (Bwanali et al., 2021). BFHI expansion was limited from 2004 due to a funding shortage and concerns about HIV in breastmilk (Health Policy Plus HP+, 2019; Kavle et al., 2019). By 2015, no hospitals were designated as baby‐friendly, but BFHI was revitalised in 2016 by the MOH with support from the USAID‐funded Maternal and Child Survival Programme (MCSP) (Kavle et al., 2019). Following MCSP's closeout in 2018, the Health Policy Plus (HP+) and Organised Network of Services for Everyone's Health (ONSE) provided training and technical support to 23 district facilities based on the BFHI 20‐hour course adapted from the WHO 2016 course and lessons learned from MCSP, resulting in 19 facilities receiving baby‐friendly certification (Health Policy Plus, 2021; Bwanali et al., 2021, personal communications, F. Bwanali of HP+ Project, March 7, 2022 and August 27, 2020; and M. Magombo of ONSE project, August 31, 2020).

## 2 METHODS

### 2.1 Case study design

This qualitative case study was conducted as two standalone data collection exercises. A holistic, multiple case design was used, where the country is the unit of analysis (Yin, 2018). The Kyrgyz Republic and Malawi were chosen based on the governments' commitment and level of implementation of BFHI activities, USAID's support for BFHI in both countries and their geographical diversity.

### 2.2 Study sites and key informant (KI) selection

In the Kyrgyz Republic, four areas were selected for study: Jalal‐Abad town and Naryn town, which received TA for BFHI from the USAID‐funded Strengthening Partnerships, Results, and Innovations in the Nutrition Globally project between 2014 and 2017, and Leilek district and Sokuluk district, which did not.

In Malawi, four sites were selected in areas that were receiving TA from USAID's implementing partners, HP+ and ONSE in the Centre and South regions. These were Dedza (Centre) and Thyolo (South) districts, which received support from HP+ and Nkhotakota (Centre) and Machinga (South) districts, which received support from ONSE.

KIs for both countries were a convenience sample based on securing a broad selection of opinions and experiences from the national, regional, district and facility levels, as appropriate.

### 2.3 Data collection

The case study included desk reviews of published and grey literature on BFHI programming, national plans and policy documents, and evidence of policy implementation specific to the selected national responsibilities and KI interviews in both countries. Local research firms were contracted to conduct the in‐depth interviews with a purposive sample of KIs, identified in consultation with the USAID Missions, the Public Health Division of the Ministry of Health (MOH) in the Kyrgyz Republic, the Department of Nutrition, HIV and AIDS (DNHA) of the MOH in Malawi and implementing partners. The interviews used open‐ended and probing questions. Interview guides were tailored to the type of respondent (Supporting Information: Annex 2 and 3). A two‐person team of an interviewer and a note‐taker conducted interviews in English or the preferred language of the KIs (Kyrgyz or Russian in the Kyrgyz Republic and Chichewa in Malawi). The researchers anonymized the recordings and interview notes, and translated interview transcripts (when applicable) before submitting them to USAID Advancing Nutrition for analysis.

In the Kyrgyz Republic, all interviews were conducted remotely due to COVID‐19. In Malawi, national‐level interviews were conducted remotely, while all other interviews were conducted in person, adhering to COVID‐19 protocols.

The research firms conducted the data collection between February 4 and April 2, 2021 in the Kyrgyz Republic and between March 8 and April 2, 2021 in Malawi.

### 2.4 Data analysis

ATLAS.ti 9 was used for coding and qualitative data analysis. A total of 38 interviews from the Kyrgyz Republic, 47 interviews from Malawi and the desk reviews from each country were coded (Supporting Information: Annex 2 and 3). The interviews from Malawi were sorted into document groups by each district, districts with reported weak or strong implementation of BFHI, implementation level (facility, zone/district, national) and respondent type. The document groups for the Kyrgyz Republic were district, implementation level and respondent type.

The findings were validated during virtual stakeholder meetings in both countries.

## 3 RESULTS

### 3.1 The Kyrgyz republic

#### 3.1.1 Breastfeeding and BFHI strategies and structures

In 2000, the MOH created the National BFHI Coordination Committee, comprised of medical and health experts including MOH specialists, heads of university paediatric departments, national BFHI trainers and Medical Accreditation Commission members. The committee has coordinated incentivizing facilities through the certification process, awarding BFHI status based on assessments, monitoring compliance to laws, reviewing promotional materials and training programmes related to breastfeeding and BFHI and adapting to breastfeeding regulations as they are updated (UNICEF & WHO, 2017).

In 2018, changes to BFHI and the health system began. The MOH reintegrated the Mandatory Health Insurance Fund (MHIF) into the Ministry. Created in 1997, the MHIF manages contracts and administers payments to health providers, monitors the quality of health services and ensures the protection of patient rights (Giuffrida et al., 2013). In 2018, the MOH began adopting the new UNICEF BFHI guidance. The Kyrgyz Republic has long prioritised breastfeeding and BFHI, and they participated in the 2017 UNICEF Compendium of Case Studies Relaying Country Experiences with BFHI, which informed the updated guidance (UNICEF & WHO, 2017). An updated national policy was enacted in 2019 (Order N1078), which shifted the focus of BFHI implementation from certification to institutionalisation. Two implementing partners said that the MOH started to roll out the updated guidance regionally, but due to a lack of trainers, funding challenges and the COVID‐19 pandemic, dissemination planning halted, with no immediate plans to resume (at the time of the study).

There is variation between written policies and policymaker descriptions of a national system for BFHI, compared to how facility directors and service providers experience BFHI implementation at the facility level. At the national level, there is a coordination committee, national policies and curricula; however, facility administrators and service providers were either not aware of these structures or found them implemented inconsistently. For example, breastfeeding training was often ad hoc in facilities, without the assistance of external trainers. Even for BFHI‐certified facilities, recertification was not taking place as scheduled. Additionally, though policymakers said that BFHI‐related indicators were integrated into health care evaluation systems and health care curricula, these changes were not mentioned by facility administrators or service providers.

The new 2019 Order N1078 is expected to better institutionalise the Ten Steps in facilities by moving away from BFHI certification and towards results‐based financing by revising training requirements and curricula and training new trainers. Due to the COVID‐19 pandemic, the roll‐out was suspended in 2020. At the time of writing, we do not have additional information about how the process will unfold.

#### 3.1.2 Responsibility #5: Incentives and sanctions

The interviews and desk review revealed no specific incentives or sanctions for BFHI in the Kyrgyz Republic. Policymakers, facility administrators and service providers stated that receiving the BFHI certification plaque to prominently display for staff and patients to see was the incentive, along with the moral obligation to support breastfeeding because of its importance for both mother and child.

‘In the beginning there was public recognition, it was published in the newspaper. Everyone would hear that this particular hospital obtained the title…’ —Policymaker

Respondents also said that previous financial sanctions imposed on health care facilities for poor‐quality medical services had lost impact on motivating health care providers to improve health care services generally.

‘MHIF applied financial sanctions for poor quality medical care for 20 years, however the numbers did not change for better and there was no interest on the part of facility administration, even if money was cut from their budget. Therefore, the MHIF has moved from sanctions to incentives. We have seen that incentives are much better, based on the results of the [World Bank] project. We started to see changes in motivation for improvement and interest’. —MHIF

In 2018, as part of monitoring activities, the MHIF began implementing a results‐based financing approach for health facilities and medical providers using a balanced scorecard. This approach was adapted from a 2014 to 2019 World Bank results‐based financing project, which piloted performance‐based payments for quality of care. The scorecard uses qualitative and quantitative indicators to assess health facility and medical provider service quality and a point system to determine payments. The only questions regarding breastfeeding pertain to qualitative interviews with discharged mothers when they are asked about their breastfeeding practices as an indication of appropriate messaging from medical providers (World Bank, 2020).

‘There is no financial incentive for getting the BFHI title… Moral incentive and publicity are the only motivations, and only apply to maternity hospitals’. —Donor

Other BFHI indicators are not collected. Although not directly related to national BFHI policies, the scorecard was mentioned by multiple respondents as a possible incentive for health workers to provide high‐quality breastfeeding counselling and support and a good way to measure the quality of support if breastfeeding indicators were included and funding resulted from high scores.

‘If we want to achieve good results, motivation must be significant. Unfortunately, government support is not enough to make this motivation significant… We cannot significantly increase the interest of employees… Small money is always better than sanctions. Sanctions today cause only a negative attitude’. —MHIF

#### 3.1.3 Sustainability

##### Budgeting and finance

Although asked about budgeting and financing related to incentives and sanctions for BFHI, respondents did not discuss how incentives or results‐based financing are specifically budgeted. Their responses focused on how financial incentives are used to motivate staff a little but as a sanction are not effective.

##### Advocacy and communication

Respondents did not describe advocacy actions for incentives and sanctions, even when asked directly. According to the interviews, international organisations engage in advocacy and communication around BFHI from the highest government level down to community‐level structures.

‘One breastfeeding commission from the MOH gave us a questionnaire, but it was only once, and we did not receive any feedback’. —Facility administrator

The MOH's Republican Centre for Health Promotion and Medical Communication also runs campaigns on breastfeeding promotion, which disseminate materials and host programmes on national television and radio, targeting remote and rural regions. Some service providers felt that advocacy around BFHI efforts through mass media would incentivize staff because families would better understand the importance of breastfeeding and have greater expectations for support at facilities.

‘It is necessary to work on creating an enabling environment starting from the highest level and ending on the community level’. —Implementing partner

##### Monitoring and evaluation

Respondents described distinct processes to monitor implementation of the Ten Steps, but not one comprehensive national monitoring system. Respondents described the MHIF‐balanced scorecard for results‐based financing as the main tool for evaluating health service quality. Points earned on the scorecard determine how much financial resources facilities receive. In some facilities, respondents said that there had previously been helpful quarterly assessments with the scorecard but that these had stopped, as had the financial awards. In other facilities, assessments were still happening, but scoring was not always transparent. One facility was doing their own scoring,

‘I do rounds and conduct surveys of mothers who gave birth the previous day—if they had consultations in prenatal departments, if the doctors consult them about the importance of breastfeeding in the maternity ward, how to do breastfeeding correctly’. —Service provider

and another respondent expressed the desire for the World Bank project to start up again, implying that the facility was receiving neither assessments nor financial awards.

‘In 2014, we had a program supported by the World Bank to support quality of service delivery to the population, and a scorecard was used to assess the performance of each hospital on a quarterly basis. Hospitals were paid based on the points they received. Forty‐three hospitals participated. The MHIF gives points according to the quality of medical care’. —Policymaker

#### 3.1.4 Barriers and challenges

The health care system in the Kyrgyz Republic is in the midst of a reform. The monitoring results‐based financing system has only recently been incorporated, and the process to adopt the new BFHI guidelines was halted due to the pandemic, which shifted country resources to the health crisis.

‘[Due to COVID‐19] Not enough time, not enough staff, sometimes no staff to train, especially after the pandemic, many doctors left their jobs. Staff members say they prefer offline training, which is more effective to understand the material’. —Policymaker/Regional facility director

‘In recent years not a single national trainer has traveled or provided training’. —Facility administrator

There is no current formalised structure to incentivize adoption of the Ten Steps in facilities. Some service providers mentioned providing breastfeeding support to mothers and infants because it is the right thing to do.

‘No rewards, no sanctions, no motivation. There should be, but there is not. No BFHI status. We just apply ourselves’. —Service provider

‘I think sanctions never motivated anyone to do better. Praise/compliments and support work better especially among young specialists in front of other specialists’. —Facility administrator

Policymakers and implementing partners reported that the only incentive for facilities to obtain BFHI status is the plaque, though respondents suggested that a lack of additional pay disincentivizes this work.

‘Firstly, this [challenge] is due to the daily work of medical staff in Kyrgyzstan. Their hard work is not financially appreciated and therefore only selfless patriots remain. But the country will hardly be able to continue working on patriotism for so long… The financial incentives for health care in Kyrgyzstan need to be revised’. —Policymaker

### 3.2 Malawi

#### 3.2.1 Breastfeeding and BFHI strategies and structures

##### National‐level coordination

Malawi has multiple structures from the national to the facility level that support and oversee BFHI. The national Infant and Young Child Feeding (IYCF) Technical Working Group (TWG) meets quarterly to oversee BFHI. The IYCF TWG is part of the national Multi‐Sector Technical Nutrition Committee (MTNC), which meets biannually to provide technical oversight of the implementation of the policy. The DNHA, which established the MTNC, is the main government institution providing policy guidance and overall coordination of nutrition activities and services, including BFHI. When resources are available, the national IYCF TWG certifies hospitals as baby‐friendly upon satisfactory completion of internal and external assessments from MOH BFHI master trainers (Kavle et al., 2019, Personal communication with Mpumulo Magombo and Elimase Kamanga, ONSE Health, May 28, 2020).

##### Zonal‐level coordination

Zones, now called quality improvement (QI) offices, are responsible for strengthening the health system. QI officers visit facilities to support them with the process and facilitate training on QI issues, including for BFHI.

##### District‐level coordination

The multisectoral District Nutrition Coordination Committee (DNCC), under the District Executive Committee, meets monthly. A BFHI task force, under the direction of the DNCC and chaired by the district BFHI coordinator, champions BFHI activities; provides technical backstopping to the district health facilities; and coordinates internal assessments, in‐service training and mentorship. The task force oversees monitoring of the Code.

##### Facility and service delivery

Health facilities implementing BFHI have an IYCF policy that covers the BFHI Ten Steps. Each facility has a BFHI coordinator, who oversees a task force responsible for enforcement of the policy. In addition to serving on the task force, the matron of the health facility and those in charge of maternity and newborn care wards and antenatal and under‐5 clinics provide supervision, monitor policy adherence and compliance with the Code and facilitate internal assessments.

#### 3.2.2 Universal coverage

Responsibility #6: TA to Facilities

As defined by respondents, TA is focused on building health worker competencies and achieving BFHI certification. TA was also described to be provided for national planning of BFHI scale up and to individual facilities that provide maternity and newborn care in preparation for BFHI certification. Respondents described TA (including one‐on‐one coaching, supervision and mentorship to reinforce breastfeeding knowledge and practical skills) as helping to overcome the challenges of staff shortages and high turnover by strengthening health professional competency and ensuring the quality of service delivery within facilities. Since 2018, TA from HP+ and ONSE, working through Malawi's structures, introduced QI approaches to improve hospital procedures and processes after an initial assessment was conducted.

‘… At the national level, HP+ meets with officers in the nutrition department… and plans with them [on] what needs to happen in… BFHI…[The] plan is taken to HP+ office, and we request funds for those activities. MOH and HP+ work together towards implementation. They monitor and evaluate together until the facility achieves BFHI’. —HP+ staff

Development partners commonly support TA through in‐service training within facilities, training of trainers, supportive supervision and providing resources (i.e., training and QI materials, supervision checklists and travel costs).

‘We are working with the Ministry on revitalization of BFHI. Malawi has been implementing it for a number of years. As ONSE, we make sure facilities with maternity clinics are implementing the Ten Steps…. We work with the BFHI task force within facilities some are active some are not. We work with them to plan the next step’. —ONSE staff

Professional associations and regulatory bodies expressed a capacity and willingness to provide TA by monitoring the quality of service delivery and identifying gaps and providing guidance if additional resources were available.

‘Each and every facility should be monitored and supervised at least twice a year, but we fail to manage due to lack of resources’. —Regulatory body

‘For now, we have not done that, we have not provided technical assistance [or] monitoring for BFHI’. —Professional association

Zone and district managers are responsible for planning TA provision to health facilities.

‘[We come] up with a plan of action [that includes] supervision, mentorship, and working hand in hand with the partners. [We make] plans for CPDs [continuing professional development] so that we can also have issues of BFHI [covered]’. —District nursing officer

The BFHI coordinator oversees TA,

‘Clinical officers, doctors, nurses—all these provide support through mentorships or coaching’. —Zone manager

Facilities are prioritised to receive TA through a variety of criteria. In some districts, all maternity facilities were prioritised. In another, the zone prioritises facilities with a strong interest in and implementation of BFHI. Some districts prioritise facilities in remote areas that are at a disadvantage. However, in one of these districts, it was reported that facilities are prioritised by their volume of work.

‘The zone prioritizes those that have a strong interest and are doing better… But at times those districts that are not doing well, there is a lack of interest or motivation and it's thereby harder to make them shine’. —Zone manager

‘The volume of work they have in the facility will be prioritised thus if there is a lot of work, it is good to import the breastfeeding knowledge’. —District nursing officer

QI circles (groups of health workers) at the department or unit level within the facility serve as a formal mechanism to improve their BFHI performance by meeting regularly to identify and solve problems. QI for BFHI is integrated with other services in antenatal, maternity and newborn care. With external support and TA from partners, QI addresses both individual and facility practices.

‘With quality improvement you don't dictate what people have to do, you make them realize that you have to do this, as it has to come out from themselves as they will be motivated’. —Zone manager

‘What is involved in the BFHI is what we regulate… It is part of pediatrics and child health care. If a facility doesn't meet the requirements to provide childcare, we enforce those standards because in BFHI we [could be] involved right from the start… but in our inspections we don't assess only for BFHI specifically’. —Regulatory body

In three districts where BFHI implementation is reportedly strong, respondents described QI to include assessing the quality of service delivery and adherence to policies and protocols at the facility level with a checklist or spot checks. If performance is found to be insufficient, the BFHI coordinator follows up with the QI team within the appropriate department to address the gaps. At the district level, linkages with other sectors and partners help to coordinate action.

‘If it can be sorted through supervision, it is corrected then and there. If it cannot be sorted then and there—the committee meets and discusses on how to resolve the issue. It can also be addressed through quality review meetings. The issues are directed to relevant authorities by the committee’. —District medical officer

There was no evidence of cross‐facility QI circles in the study areas, but a respondent suggested that such cross‐facility TA would be useful. A respondent at a district hospital that was certified 4 years ago said that QI is a new approach, and currently, the teams are not vibrant due to a shifting focus away from BFHI by the implementing partner.

‘At the facility, quality improvement [for BFHI] hasn't been the main focus because [the] priority is neonatal improvement’. —District nursing officer

This was further exacerbated by the pandemic.

#### 3.2.3 Sustainability

##### Budgeting and finance

Respondents were asked about budgeting and finance for TA. Their responses focused on overall budgeting for BFHI and nutrition.

‘Engage partners to support them to reach other health centers where training has not been done’.—District management

‘The district implementation plans still include BFHI, [using] local resources at the district level’. —District hospital staff

They did note that development partners (who support and provide TA) do not always have funds for BFHI‐related activities because of competing priorities for other nutrition and health funding.

‘The ministry and the partners MSH [Management Sciences for Health] (ONSE) should be addressed but their focus is maternal and neonatal health…’ —Service provider

‘There's a good vibrant team. At the facility, members of staff are eager to see the program does not end, especially midwives. The policy is still available and structures of BFHI—this will make continuation easy. The new government is a listening government. If partners would come in, implementation would be easy’. —District health management team member

##### Advocacy and communication

In Malawi, policymakers, professional bodies and service providers are aware of the need for advocacy at multiple levels to fully institutionalise BFHI. Respondents recommended advocacy with district‐level governance structures (the District Council) so that funds are allocated at this level and not just nationally.

‘…Advocacy should start at the central level, because I don't think in the budgeting there is a separate line special for breastfeeding, so breastfeeding has been incorporated into…maternal health… It should have its own funding… and maybe to achieve that we need to involve stakeholders that are involved in breastfeeding issues, so those ones can help to make sure that there is enough support give toward breastfeeding’. —International stakeholder

They also called for advocacy with the community to ensure a continuum of care. Respondents did not describe specific advocacy actions for TA, beyond general funding and resources for BFHI.

##### Monitoring and evaluation

The desk review revealed protocols and assessment tools for maternal, newborn and child services and quality assurance that include aspects of BFHI. For example, the MOH checklist for quality assurance and standard protocols for managing labour, delivery and newborn care includes early initiation of breastfeeding within 30 min, skin‐to‐skin and rooming in. However, systematic measurement and reporting of those indicators were not described.

‘At national level there is only one indicator (initiation of breastfeeding). We can get some more indicators from the Ten Steps’. —Facility administrator

Data on process indicators or achievements are not systematically collected or shared at the district and the national level. For example, the MOH does not routinely collect data on the number of the health workers trained or monitor BFHI adherence, and they conduct external assessments periodically depending on external funding. Rather, partners supporting these activities collect the data for their programme purposes. Respondents suggested collecting outcome data to determine impact.

‘The key indicator should be the outcomes not the processes, not the number of trainings conducted; for example I would like to see the proportion of newborn[s] that are initiated to the breast in the first 30 min or one hour of birth, the proportion of mothers who have been counseled or supported, etc. … People can be trained but … not everybody implements’. —International stakeholder

#### 3.2.4 Barriers and challenges

A lack of resources—financial and human—was a common challenge. Most of the funding comes from development partners for specific activities, which is insufficient to support universal coverage of BFHI implementation (i.e., a goal of training 30,000 health workers).

‘Funding shortage of transport affects delegated members to supervise, monitor, and provide technical backstopping to health facilities in their districts—as they fail to go and supervise the communities. Morale for BFHI implementation is low—due to lack of training due to financial problems’. —Facility administrator

‘People expect the nurses and midwives to do everything. The same midwives should do deliveries, conduct training and awareness in BFHI—so they get overwhelmed and they just end up prioritizing saving lives. This is due to shortage of human resource[s]’. —Regulatory body

However, the COVID‐19 pandemic diverted resources to the response efforts, and group meetings and assessments were cancelled. Staff reported feeling overwhelmed due to staffing shortages and reassignments due to COVID‐19.

‘…The nurses that have been trained provide better care than those who have never had training … it was evident when the experienced nurses were shifted to working COVID wards…’ —District medical officer

‘…funding is channeled to prevention of COVID‐19, as such periodic assessments of most programs have been put on hold’. —District nursing officer

Although Malawi operates under a decentralised system and structures have devolved from the national to facility level to support BFHI, they appear to be mainly coordinated at the national level, which limits districts' ability to adequately oversee BFHI implementation.

‘…When there is an issue on BFHI, it takes time, as we have to go through the [national] desk officer or refer issues through them’. —Zone manager

## 4 DISCUSSION

The new BFHI guidance describes nine national responsibilities to expand the coverage and sustainability of the Ten Steps (UNICEF & WHO, 2018). This paper describes progress in two of the nine responsibilities (incentives and sanctions and TA along with efforts in financing, advocacy and monitoring needed to sustain them) in two different country contexts. Using a qualitative approach, perspectives from a cross section of policymakers, health professionals, managers at various levels of the health system and stakeholder organisations were analysed to understand the progress made to date. The study was not meant to be a critical review or evaluation of the countries' BFHI programmes.

The Kyrgyz Republic has not yet identified the most appropriate incentive or sanction to implement relevant to BFHI. Respondents clearly indicated that financial sanctions that were in place for general hospital care before 2018 were not effective and so were not considered a viable option for BFHI under the new guidance. Despite the 2019 national policy halting certification plaques for BFHI, incentives like moral responsibility and baby‐friendly designation continued to resonate with respondents as a motivator for facilities and providers to comply with the Ten Steps. The recommended shift in the guidance may take some time to achieve, as the baby‐friendly hospital designation continues to be deeply valued by providers. The new BFHI guidance recommends that countries who are already applying performance‐based financing for other relevant interventions may consider this as an incentive option. While not intended to focus on the Ten Steps, the World Bank project in the Kyrgyz Republic demonstrated that performance‐based financing or balanced scorecards may improve aspects of quality of care but was not optimal for improving clinical practices—and there are no results reported on the effect on breastfeeding support (Gergen et al., 2017; World Bank, 2020). Although the 2019 national policy intends to institutionalise BFHI through performance‐based financing, this was not yet occurring at the time of this case study. The Kyrgyz Republic MHIF scorecard evaluation system only includes indicators related to breastfeeding practices after discharge. To fully integrate compliance with all Ten Steps, the national policies related to BFHI and MHIF would need to be harmonised. While the use of performance‐based financing exists in many low‐ and middle‐income countries (LMIC), including for maternity care services (Gergen et al., 2018), a review of 68 quality checklists used in 28 countries documented a lack of indicators related to the postpartum period (Josephson et al., 2017). This lack of documentation of breastfeeding‐related postpartum indicators in the literature of performance‐based financing programmes makes it difficult for a country to adopt this approach as ‘evidence‐based’ without further evidence from implementation research that specifically reports on breastfeeding outcomes. To develop and implement strong incentives for BFHI in the Kyrgyz Republic, further research on provider motivations and potential adaptations to the MHIF scorecard evaluation systems is needed.

While many studies have identified gaps related to breastfeeding or BFHI in LMICs, there is less documentation on how TA has helped address existing gaps (Agbozo et al., 2020; Kinshella et al., 2021; Nyondo‐Mipando et al., 2021). Results from Malawi demonstrate that TA is primarily used for competency building, including training, coaching, mentorship and supervision. While training is an important component, countries should consider other areas that TA could benefit, particularly around monitoring and data management. One systematic review of health facility barriers and facilitators for breastfeeding suggested that areas such as health facility infrastructure, supplies and staffing could also benefit from TA (Kinshella et al., 2021). Partners supporting TA introduced QI processes for the Ten Steps. Although QI approaches are common in LMICs, documentation of their use for BFHI is not (Tamburlini et al., 2020; Tibeihaho et al., 2021; Valente et al., 2021). In Spain and the United States, the use of QI collaboratives, which are groups of facilities learning together and providing support to one another through QI processes, has demonstrated positive results for increasing compliance with BFHI (García‐de‐León‐González et al., 2011; Grossniklaus et al., 2017). Since facilities in Malawi have received TA to utilise QI methods for BFHI, the use of collaboratives could be explored once the pandemic restrictions are lifted on movement. An overarching plan for prioritising facilities to be provided TA could not be discerned based on the varied strategies described by KIs. Determining which strategy or strategies are most successful to reach the most facilities with TA to achieve baby‐friendly status could help scale up coverage in Malawi. One interesting finding was that local professional associations and regulatory bodies reported that they would be interested in providing TA on BFHI, if they had resources to do so. Given their roles as regulatory bodies and professional organisations, and in line with the 2018 implementation guidance, these organisations may be best suited to work with the MOH with regard to external assessment, a responsibility that we did not explore as part of this study.

Overall, this study found strong policy environments and mechanisms with the potential to scale up and sustain BFHI in both countries. However, financial and human resources are further constrained by the COVID‐19 pandemic. The responsibilities required to sustain BFHI (i.e., financing, communication and advocacy and national monitoring) are needed to sustain universal coverage actions as a whole. So, needs for specific universal coverage responsibilities were difficult to tease out. Support is still required in both countries for scaling up and sustaining implementation of the Ten Steps, as documented in other countries (Agbozo et al., 2020). The case studies identified local resources, such as professional associations and regulatory bodies that should also be considered in the future as key TA providers for various aspects of BFHI, though some funding for these entities would be required.

Tools and resources are available that could be used to help countries assess these national responsibilities in greater depth, like the World Breastfeeding Trends Initiative (WBTi) tool or the Becoming Breastfeeding Friendly (BBF) Index, though these extend beyond BFHI implementation and explore national implementation or readiness for breastfeeding promotion, protection and support programming generally (BPNI and IBFAN, 2019; Perez‐Escamilla et al., 2012). The WBTi and BBF processes were successfully used in many countries with an aim to assess the status of national policies and programming for breastfeeding generally (Aryeetey et al., 2018; Gupta et al., 2020). WHO and UNICEF are developing internal and external assessment tools that may further assist countries with some of the national responsibilities.

There were some limitations of this case study. First, due to the timing of the release of the new BFHI implementation guidance in late 2018, and the onset of the COVID‐19 pandemic shortly after, and as our results documented, this external factor may have impacted countries' ability to take further action on and implement these national responsibilities. Therefore, this limits the applicability of the findings to other countries who may attempt to adopt these responsibilities. Additionally, due to COVID‐19, the study designers could not collect the data directly. Deeper probes may have been missed, which could have resulted in richer findings and understanding of the context of each responsibility. However, local data collectors who spoke the language also presented an advantage. Nuances in perceptions and opinions may have been lost in translation of data, but given the wide array of informants and responses and the validation meetings, we were able to triangulate the data and overcome those limitations. This case study represents views of specific stakeholders in both countries, and while care was taken to select a diverse group of KIs, other opinions or critical aspects may not be reflected. A strength of this case study is that it documents both successful and unsuccessful approaches related to the national responsibilities of sanctions and incentives and TA. While the implementation of the management and clinical steps are critical to document, these national responsibilities comprise the enabling environment and require sharing lessons learned to improve the quality of care for mothers and newborns.

## 5 CONCLUSION

As WHO and UNICEF no longer require facility designation to become baby‐friendly, countries are scrambling with limited resources to find ways to address the national responsibilities, which are all interconnected and critical to universal coverage and sustainability. They are often choosing to maintain the certification process to some extent to achieve compliance with the Ten Steps. TA is commonly provided for competency building and supportive supervision while awaiting funding for in‐service training, though other aspects of the change process could benefit from focused TA. For countries considering incentives and sanctions, behavioural research to understand motivations for both providers and the health system, as well as adopting and monitoring hospital‐related postpartum process indicators, is necessary to understand what options for incentives or sanctions may be most effective. To institutionalise BFHI, policies need to be supported by national and subnational plans and coordination and should be effectively communicated throughout the health system. These plans are also needed for creating a strategy to extend TA to a broader number of facilities by working with groups of facilities to implement the Ten Steps. More multisectoral advocacy and communication is needed to ensure awareness of BFHI policies across and beyond the health system and to secure national and local government commitment to funding BFHI within and beyond the MOH, especially in contexts constrained by human and financial resources. Additional sharing of programmatic experiences and tools related to the national responsibilities, particularly in the LMIC context, is needed to help countries fully integrate BFHI into standards of care to reach universal coverage and sustainability.

## AUTHOR CONTRIBUTIONS

Altrena Mukuria‐Ashe and Jeniece Alvey designed the study with USAID support; Alyssa Klein and Charlotte Block adapted the design for the Kyrgyz Republic, oversaw data collection, conducted data analysis and wrote sections on the Kyrgyz Republic. Adil Mansimov and Samat Okenov collected data, conducted high‐level analysis and draughted an overview of Kyrgyz Republic results. Kanji Nyambo oversaw data collection with George Mtengowadula and Godwin Nyirongo in Malawi. Altrena Mukuria‐Ashe, Kanji Nyambo and Malia Uyehara conducted data analysis on Malawi and draughted sections of the manuscript. George Mtengowadula and Godwin Nyirongo conducted high‐level analysis and draughted an overview of Malawi results. Jeniece Alvey reviewed and draughted sections of the full manuscript.

### CONFLICT OF INTEREST

Jeniece Alvey is employed through the USAID funded Global Health Technical Professionals (GHTP) mechanism and is employed by one of the implementers, The Public Health Institute. The opinions herein are those of the authors and do not necessarily reflect the views of the US Agency for International Development or the US Government or the Public Health Institute.

## ETHICS STATEMENT

The JSI Institutional Review Board (IRB) determined the study to be exempt from human subject research oversight. The IRBs of the American University of Central Asia, Kyrgyz Republic and the National Health Science Research Committee, Malawi, approved the respective studies. All participants provided written or verbal informed consent for participation and for recording the interview. Declining to record the interview did not affect participation.

## Supporting information

Supporting information.

Supporting information.

Supporting information.

## Data Availability

The data are not publicly available due to privacy or ethical restrictions.
