# Supplementary material for: Implementing two national responsibilities of the revised UNICEF/WHO Baby‐Friendly Hospital Initiative: A two‐country case study
Source: Matern Child Nutr. 2022 Sep 29;19(1):e13422. doi: 10.1111/mcn.13422 (PMC9749588; doi:10.1111/mcn.13422)
Supplement: Supplementary file 1 — Supporting information. [file MCN-19-e13422-s001.docx]

Annex 1. Nine Key National Responsibilities for BFHI

| **Brief Description of National Responsibility** | **Objective** |
| --- | --- |
| **Responsibility #1: National leadership and coordination** requires the establishment of a multi-sectoral national coordination body responsible for the protection, promotion, and support of breastfeeding, especially in facilities providing antenatal, maternity, newborn care services. The coordinating body should have a term of reference and strategic plan, coordinate all key functions of the national BFHI program, and appoint a focal person to oversee support of breastfeeding in facilities. | Universal coverage |
| **Responsibility #2: Policies and professional standards of care** integrate the Ten Steps into relevant national policy documents and professional standards of care. Through regulation, accreditation, or certification, governments should require healthcare facilities to adhere to policies and procedures that promote, protect, and support breastfeeding. | Universal coverage |
| **Responsibility #3*: Health professional competency building** ensures health professionals and managers can implement the Ten Steps so breastfeeding is protected, promoted, and supported in facilities that provide maternity and newborn services. This responsibility includes adapting, updating, or developing curricula and materials; providing designated teaching staff with appropriate qualifications and experience; and carrying out training for all professionals interacting with pregnant women, deliveries, and newborns. The training includes pre-service, continuing education, and in-service training. Facilities are responsible for assessing competencies and ensuring their staff have the knowledge and skills they need (WHO and UNICEF, 2018; 2020). | Universal coverage |
| **Responsibility #4: External Assessment** utilizes systems to, regularly, evaluate adherence to the Ten Steps. All facilities providing maternity and newborn services are responsible for providing care for mothers and newborns according to Baby-Friendly guidelines. | Universal coverage |
| **Responsibility #5*: Incentives and sanctions** are used to motivate health facilities to comply with the Ten Steps. Incentives and sanctions may vary in public and private facilities or country contexts. Examples of incentives include financing that increases or decreases with a facility’s level of compliance with BFHI standards; performance contracts for health professionals and managers that require meeting certain goals; public acknowledgement of excellence; and public reporting of facilities’ indicators and outcomes (WHO and UNICEF, 2018). | Universal coverage |
| **Responsibility #6*: Technical assistance to facilities** is provided by experts who have managed the change process in other facilities to adopt the Ten Steps as standard of care. Technical assistance to reach universal coverage is resource- and time-intensive, so countries are encouraged to develop a cadre of technical assistance professionals. Countries may choose to form collaboratives to mutually support policy and practice change. If resources are limited, countries may prioritize certain facilities to receive technical assistance first (UNICEF and WHO, 2018). | Universal coverage |
| **Responsibility #7: National monitoring** of the implementation of the Baby-Friendly Hospital Initiative. Countries need to monitor their BFHI activities, breastfeeding outcomes, and clinical practices at the national and subnational levels. | Sustainability |
| **Responsibility #8: Communications and advocacy** to advocate for BFHI with relevant audiences. The national coordination body will identify key audiences, and conduct communications and advocacy efforts with them to ensure sustainability of BFHI implementation (e.g., facility leaders, professional associations, legislators, communities and others important for breastfeeding programming and BFHI implementation). | Sustainability |
| **Responsibility #9: Identify and allocate financing** to ensure the ongoing funding of BFHI. Incorporate BFHI activities into regular government budgeting processes in a sustainable way. | Sustainability |

*Implementation of this responsibility was explored in this case study.

Adapted from UNICEF and WHO, 2018, p. 25
