# Supplementary material for: Implementing two national responsibilities of the revised UNICEF/WHO Baby‐Friendly Hospital Initiative: A two‐country case study
Source: Matern Child Nutr. 2022 Sep 29;19(1):e13422. doi: 10.1111/mcn.13422 (PMC9749588; doi:10.1111/mcn.13422)
Supplement: Supplementary file 2 — Supporting information. [file MCN-19-e13422-s003.docx]

Annex 2. Sampling of Respondents by Category in Malawi

| **Guide #** | **Participants** | **National** | **Zones** | **Districts** | **Facility** | **Subtotal** |
| --- | --- | --- | --- | --- | --- | --- |
| II, III | **Policymakers:**  MOH Directorate (IYCF, quality management, DNHA, finance, and others)  **Regulatory bodies:**  Medical Council of Malawi and Association of Malawi Midwives | 6 | -- | -- | -- | *6* |
| IV, V | **Heads of health professional training institutions:**  Nurse midwives, medical doctors, and others  **Professional associations:**  Nurses and Midwives, Pediatric and Nutrition Societies and Associations | 3 | -- | -- | -- | *3* |
| VI | **Stakeholders:**  WHO, UNICEF, USAID, Christian Health Association of Malawi, Save the Children | 6 | -- | -- | -- | *6* |
| VII | **Zonal and district managers:**  District Health Management Team (four districts) and DNCC | -- | 5 | 6 | -- | *11* |
| VIII | **Facility administrators/program managers** (four facilities):  Medical officer and coordinators (BFHI, Safe Motherhood, Kangaroo Mothercare) | -- | -- | -- | 9 | *9* |
| IX | Service providers (four facilities)  Antenatal, maternity/postnatal, newborn care | -- | -- | -- | 12 | *12* |
|  | **Totals** | **15** | **5** | **6** | **21** | ***47*** |
