# Supplementary material for: Implementing two national responsibilities of the revised UNICEF/WHO Baby‐Friendly Hospital Initiative: A two‐country case study
Source: Matern Child Nutr. 2022 Sep 29;19(1):e13422. doi: 10.1111/mcn.13422 (PMC9749588; doi:10.1111/mcn.13422)
Supplement: Supplementary file 3 — Supporting information. [file MCN-19-e13422-s002.docx]

Annex 3. Sampling of Respondents by Category in the Kyrgyz Republic

| **Guide #** | **Participants** | **National** | **Regional** | **Facility** | **Sub-**  **total** |
| --- | --- | --- | --- | --- | --- |
| II | **Policymakers:**  MOH (maternal and child health, nutrition), National Coordinating Committee for BFHI, etc.  **Professional associations** (pediatrics, obstetrician- gynecologist, nurses, nurse midwives)  **Mandatory Health Insurance Fund** | 10 | -- | -- | *10* |
| III | **Regulatory bodies** | 2 | -- | -- | *2* |
| IV | **Heads of professional associations** | 2 | -- | -- | *2* |
| V | **Heads of health professional training institutions** | 6 | -- | -- | *6* |
| VI | **Stakeholders**: **(**WHO, UNICEF, USAID, *Deutsche Gesellschaft für Internationale Zusammenarbeit*, Management Sciences for Health, others supporting BFHI in country) | -- | 1 | -- | *1* |
| VII | **Facility administrators and program managers** (four facilities) | -- | 1 | -- | *1* |
| VIII | **Service providers** (four facilities)  Antenatal, child welfare, maternity | -- | -- | 15 | *15* |
|  | **Totals** | **22** | **1** | **15** | ***38*** |
